# Frequency of regular walking among Croatian adults

**DOI:** 10.2478/aiht-2024-75-3808

**Published:** 2024-03-29

**Authors:** Slaven Krtalić, Helena Križan, Sanja Musić Milanović

**Affiliations:** Croatian Institute of Public Health, Zagreb, Croatia; University of Zagreb School of Medicine, School of Public Health Andrija Štampar, Zagreb, Croatia

**Keywords:** chronic non-communicable diseases, demographics, EHIS-PAQ, health, physical activity, EHIS-PAQ, kronične nezarazne bolesti, sociodemografski čimbenici, tjelesna aktivnost, zdravlje

## Abstract

The aim of this study was to determine the share of Croatian adults who walk 210 min or more a week and to explore the relationship between regular walking and demographic factors, health-related behaviours, and chronic non-communicable diseases/conditions. To this end, we used the EHIS-PAQ questionnaire and collected self-reported data on minutes spent walking during a typical week from a total of 3,496 respondents. The data were additionally analysed by gender, age, education, residence (urban/rural), counties and regions, smoking, other types of physical activity, and diseases/chronic conditions. The results show that, overall, 40.9 % of the adult Croatian population walks 210 or more minutes a week, with the largest share found among those from the Lika-Senj County (76.8 %), those who spend 300 min or more weekly on health-enhancing (non-work-related) aerobic physical activity (57.6 %), those who reported having diabetes (49.3 %), and those aged 65–74 years (44.7 %). Despite its limitations, our study gives a valuable insight into the frequency and factors determining healthy walking habits in a representative sample of Croatian adults and provides grounds for further research.

## Abstract (hr)

Na temelju rezultata Europske zdravstvene ankete, cilj ovoga rada bio je utvrditi udio osoba starijih od 18 godina u Republici Hrvatskoj koje hodaju 210 i više minuta tjedno te istražiti povezanost redovitoga hodanja sa sociodemografskim čimbenicima, zdravstvenim ponašanjima i kroničnim nezaraznim bolestima/stanjima. Pomoću EHIS-PAQ upitnika prikupljeni su podatci o količini hodanja odraslih osoba tijekom uobičajenog tjedna u minutama. Podatci su dodatno analizirani prema spolu, dobi, obrazovanju, urbanizaciji, županijama i regijama, (ne)pušenju, drugim oblicima tjelesne aktivnosti te prema bolestima/kroničnim stanjima. Rezultati su pokazali kako ukupno 40,9 % odrasle populacije hoda 210 ili više minuta tjedno. Najveći udjeli populacije koja hoda 210 i više minuta tjedno zabilježeni su u Ličko-senjskoj županiji (76,8 %), među onima koji provode 300 i više minuta tjedno u zdravstveno usmjerenoj aerobnoj tjelesnoj aktivnosti izvan posla (57,6 %), onima koji su izvijestili da imaju šećernu bolest (49,3 %) te onima koji imaju između 65 i 74 godine (44,7 %). Unatoč svojim ograničenjima, naše istraživanje pruža vrijedan uvid u učestalost i čimbenike koji određuju zdrave šetačke navike na reprezentativnom uzorku odraslih Hrvata te pruža temelj za daljnja istraživanja.

The aetiology of chronic non-communicable diseases (NCDs) is multifactorial and complex, but they are predominantly determined by lifestyle and poor health-related habits (1). According to the World Health Organization (WHO), NCDs are responsible for 74 % of the total number of deaths each year (2), and insufficient physical activity is the fourth leading risk factor for premature mortality, accounting for about 5.3 million deaths a year worldwide (3,4,5). Even though physical activity has increased significantly over the last decades, one third of adults are not active enough to preserve health (4, 6).

In response to the need to prevent and control diseases and promote a healthier lifestyle, recent literature emphasises the health benefits of physical activity that help reduce the risk of some of the leading diseases, such as type 2 diabetes, cardiovascular disease, hypertension, stroke, obesity, various types of tumours, depression, and dementia (7,8,9). In addition, several studies have established a positive association between physical activity and life expectancy, with an estimated increase of up to seven years and a drop in premature mortality by 20–40 % (10, 11). The latest WHO report (12) shows that 150 min of moderate physical activity per week can prevent more than 10,000 premature deaths among people aged 30–70 every year, prolong the life expectancy of insufficiently active individuals by 7.5 months, and can save the European Union (EU) member states a total of eight billion euro per year in healthcare expenses.

Numerous extensive cohort studies consistently show that physically inactive adults run a significantly higher risk of premature death than the active ones (13), that increasing physical activity by one hour per week reduces the risk of premature mortality by 4 % (14), and that this can be achieved without adverse health effects, even if physical activity is vigorous (15).

Furthermore, regular physical activity can reduce symptoms and comorbidities and improve health-related quality of life in more than 25 different NCDs (16). Current evidence supports an association between greater physical activity and lower mortality rates in five specific NCDs: type 2 diabetes, hypertension, breast cancer, colon cancer, and prostate cancer (17). These health benefits of physical activity are recognised and proven for all age groups and depend on the amount, type, and intensity of physical activity (13).

According to a number of epidemiological studies (6, 12, 13), significant health benefits can be achieved with at least 150 min of moderate aerobic physical activity per week or with at least 75 min of vigorous activity per week. Furthermore, physical activity has a substantial positive impact on mental health, quality of life, sleep, stress, relationships, and socialisation (18). Even low-intensity physical activity can be beneficial against health outcomes such as adiposity, lipid and glucose metabolism, cardiometabolic health, and mortality (19). In fact, replacing sedentary time with physical activity of any intensity has shown health benefits (5, 6, 15, 20, 21, 22).

Current research of how much physical activity is enough to keep diseases at bay (23) can serve as a foundation for improved monitoring and crafting new health policies to mitigate the burden of chronic NCDs (24, 25). In that sense, the European Health Interview Survey (EHIS) is pivotal in public health surveillance to provide data on the prevalence and trends in physical activity, facilitating comparison between the EU countries. It first relied on a modified version of the International Physical Activity Questionnaire – Short Form but was then developed into the Physical Activity Level Assessment Questionnaire (EHIS-PAQ) (26). This standardised concise and specific questionnaire assesses the indicators of overall weekly physical activity and provides compelling data and useful tools to design and implement public health policies.

Relying on the results from the latest EHIS wave, we aimed to determine the share of adults who walk 210 min or more a week and explore the relationship between regular walking and demographic factors, health-related behaviours, and chronic NCDs in Croatia.

## PARTICIPANTS AND METHODS

This study relies on data gathered as part of the third EHIS cycle (EHIS 3) that took place in 2019 all over the EU and included Croatian participants. Detailed description has been published elsewhere (27). The survey was carried out by the Croatian Institute of Public Health in collaboration with local health centres, county institutes of public health, the Central Bureau of Statistics, and the Ministry of Health, following the standardised EHIS protocol.

The target population was all persons aged 15 and over, and the random sample included 3,600 households, of which 75 % (5,461 respondents) participated in the survey.

We then excluded respondents under the age of 18 and those who had someone close answer the questions on their behalf. Additionally, we excluded all respondents who had difficulty or could not walk 500 m on level ground without the use of aid and those who did not provide this information. Therefore, the final sample of 3,496 respondents consisted only of those who were 18 years of age and older, who answered the questions independently, and who had no difficulty walking 500 m on level ground without the use of any aid.

The EHIS-PAQ questionnaire was used to collect data on minutes spent walking over a typical week. Respondents were first asked to specify how many days a week they continuously walked for at least 10 min to get to and from places (such as work, store, or market) by choosing on a range between zero and seven days. Those who reported walking from 1 to 7 days a week were then asked to specify how much time they spent walking on the days they walked by choosing between the following options: 10–29 min, 30–59, 1 h to 1 h and 59 min, 2 h to 2 h and 59 min, or 3 h or more.

The total weekly walking time was then calculated by multiplying the number of walking days by the mid-range number of reported minutes as follows: 10–29 min was set to 20 min, 30–59 min to 45 min, 60–119 min to 90 min, 120–179 min to 150 min, and ≥180 min to 210 min. From this, we established the share of adult respondents who walked ≥210 min a week (≥30 min a day in average) for the entire sample and for individual subgroups.

The respondents were then grouped by gender (male and female), age (18–24, 25–34, 35–44, 45–54, 55–64, 65–74, and 75 years or older), and body mass index (BMI) based on reported height and weight (<30 and ≥30).

The level of education was based on the 2011 International Standard Classification of Education (ISCED) (28) as follows: lower (ISCED levels 0–2 corresponding to elementary or lower), medium (ISCED levels 3 and 4 corresponding to secondary or vocational), and higher education (ISCED levels 5–8 corresponding to tertiary education, bachelor level or higher).

The respondents were also categorised by residence in terms of population density (cities, towns/suburbs, rural areas) and region (Pannonian Croatia, Adriatic Croatia, City of Zagreb, Northern Croatia).

In regards to health-related behaviours, respondents were asked if they smoked tobacco (excluding electronic cigarettes or similar electronic devices) daily, occasionally, or not at all and whether their work, paid or unpaid, involved “mostly sitting or standing”, “mostly walking or tasks of moderate physical effort”, “mostly heavy labour or physically demanding work” or were “not performing any working tasks”.

Furthermore, respondents were asked to specify how much time and how many days a week they spent cycling for at least 10 min and doing sports, fitness or recreational (leisure) physical activities that increased their breathing or heart rate for at least 10 min. This information was used to calculate the average minutes spent on health-enhancing (non-work-related) aerobic physical activity a week, and the respondents were subsequently categorised as follows: not performing aerobic activities, <60 min, 60–150 min, 150–300 min, ≥300 min.

As for NCDs, obesity, and other chronic conditions, respondents were asked to report if they had any of the following in the past 12 months: asthma (including allergic asthma), chronic bronchitis, chronic obstructive pulmonary disease or emphysema, heart attack (myocardial infarction) or chronic consequences of myocardial infarction, coronary heart disease or angina pectoris, high blood pressure (hypertension), stroke (cerebral haemorrhage, cerebral thrombosis) or chronic effects of stroke, arthrosis (excluding arthritis), low back disorder or other chronic back defect, neck disorder or other chronic neck defect, diabetes, allergy (such as rhinitis, eye inflammation, dermatitis, food allergy or other excluding allergic asthma), cirrhosis of the liver, urinary incontinence or problems in controlling the bladder, kidney problems, depression, and high blood lipids. Respondents were categorised based on the number of diseases/chronic conditions into groups ranging from none to four or more diseases/chronic conditions.

### Statistical analysis

All statistical analyses were run on SPSS version 21 (SPSS Inc., Chicago, IL, USA). Differences in the share of respondents who walk ≥210 min a week between groups were analysed with the Pearson’s chi-squared test. Associations between walking ≥210 min a week and covariates were identified with binary logistic regression. The strength of associations is expressed as odds ratio (OR) and 95 % confidence interval (CI). All analyses were done on weighted data to ensure representativeness.

## RESULTS AND DISCUSSION

Table 1 shows that only 40.9 % of respondents reported walking ≥210 min a week. Their share differs significantly (p<0.001) between each subgroup, but the differences between subgroups are quite small. Regardless of the group, the share of those walking ≥210 min a week is below 50 %, which raises a red flag and calls for urgent public health action to raise awareness of the benefits of walking to preserve and improve health.

**Table 1:** Share of adults walking ≥210 min a week by demographic characteristics

	**Share of population walking ≥210 min/week**
Sex^*^	
Men	44.6 %
Women	38.0 %
Age^*^	
18–24	41.5 %
25–34	42.5 %
35–44	40.9 %
45–54	37.5 %
55–64	40.2 %
65–74	44.7 %
75+	42.7 %
Residence by population density^*^	
Cities	38.8 %
Towns and suburbs	42.5 %
Rural areas	41.9 %
Education level^*^	
Low	39.5 %
Medium	41.9 %
High	40.1 %
NUTS-2 regions^*^	
Pannonian Croatia	44.4 %
Adriatic Croatia	42.9 %
City of Zagreb	38.3 %
Northern Croatia	34.9 %
**Total**	**40.9 %**

* p<0.001.

NUTS-2 – non-administrative regions as defined by the Eurostat’s Nomenclature of Territorial Units for Statistics of the European Union (available at: https://ec.europa.eu/eurostat/web/nuts)

Fewer women seem to be walking ≥210 min a week than men, and although this finding is consistent with previous research on overall physical activity on the global level (29), there is little evidence of gender differences in walking alone (30).

**Figure 1:**
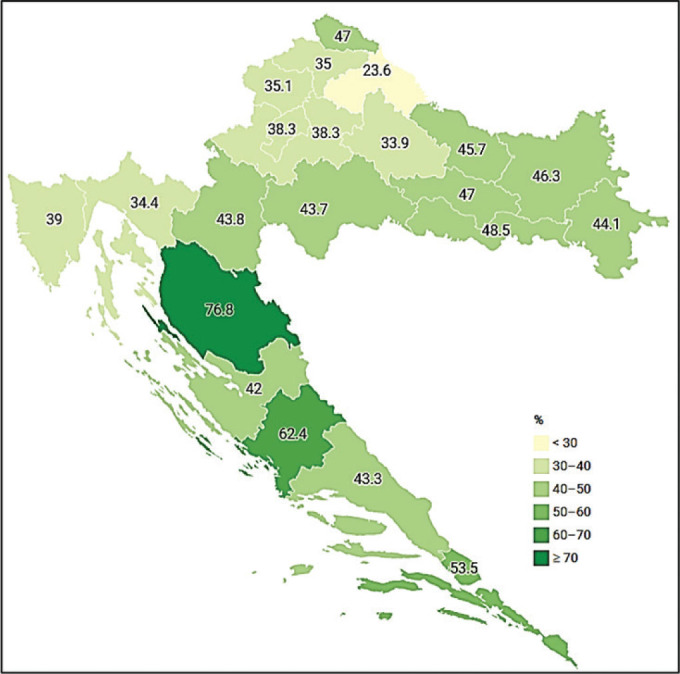
Share of Croatian adults walking ≥210 min a week by county (see detailed map of Croatian counties at *https://croatia.eu/index.php?view=article&id=30&lang=2*)

As for age, the highest share was reported by 65–74-year-olds (44.7 %), which suggests that retired people in Croatia engage in regular walking as their preferred form of physical activity, which is in line with the rest of the EU (31). Regional analysis shows that residents of the Pannonian region have the largest share of 210+ min walkers (44.4 %). These findings are different from those reported in the National Traffic Development Strategy (32), claiming that residents of the Adriatic Croatia make 60 % more trips on foot than residents from the continental Croatia. Of course, these differences may be owed to different research criteria and methodology, as the national strategy does not take into account the duration of walking. If we go deeper into regions, our findings single out the residents of Lika-Senj County as having the highest share of 210+ min walkers (76.8 %) (Figure 1). This is the largest and least populated county with the least developed public transport network and the fewest registered cars in Croatia (33,34,35). Therefore, its residents are quite likely to cover greater distances on foot every day than the residents of other counties. Furthermore, Lika-Senj County has the largest 65+ population (36), for which we have already established to have more 210+ min walkers than other age groups.

In addition, city residents seem to have the lowest share of 210+ min walkers (38.8 %), which again may reflect much higher availability of public transport and fast-paced lifestyle as opposed to towns/suburbs and rural areas.

Although research of overall physical activity suggests that those with higher education are more physically active (30), our findings single out respondents with secondary education as having the highest share of 210+ min walkers (41.9 %), whereas those with lower education have the lowest share (39.5 %). It seems that high education in our sample entails less free time for walking despite better awareness of its health benefits.

Table 2 shows the share of the population walking ≥210 min a week in different subgroups of health-related behaviour. The highest share is reported by those who do mostly labour-intense, physically demanding work (51.5 %) and the lowest by those who have no working tasks (25.0 %). This pattern is repeated with respondents grouped by time spent on non-work-related physical activity, as those who spend the most time on them report the highest share of 210+ min walkers (57.6 %), and those who spend the least time report the lowest share (35.5 %). Historically, studies of the relationship between occupational and non-occupational physical activity have been guided by two theories: compensation and spillover (37). The compensation theory suggests that high physical exertion at work makes one less likely to do physical activity outside of work, while the spillover theory suggests that high physical activity at work makes one more likely to continue with it outside of work (38). Our findings obviously speak in favour of the latter.

**Table 2:** Share of adults walking ≥210 min a week by health-related behaviour

	**Share of population walking ≥210 min/week**
Smoking^*^	
Daily smoking	42.4 %
Occasional smoking	34.3 %
No smoking	40.8 %
Work-related physical activity^*^	
Mostly sitting or standing	29.0 %
Mostly walking or tasks of moderate physical effort	45.7 %
Mostly heavy labour or physically demanding work	51.5 %
Not performing any working tasks	25.0 %
Time spent on health-enhancing (non-work-related) aerobic physical activity^*^	
None	35.5 %
<60 min	37.5 %
60–149 min	39.3 %
150–299 min	47.7 %
≥300 min	57.6 %

* p<0.001

In regards to smoking, we see no clear pattern, as the lowest share of 210+ min walkers is reported by occasional smokers (34.3 %), a little higher by non-smokers (40.8 %), and the highest by daily smokers (42.4 %).

Table 3 compares the share of adults walking ≥210 min a week between respondents with and without specific self-reported diseases/chronic conditions in the past 12 months. Differences that stand out are those between respondents with and without liver cirrhosis (15.6 % vs 41.1 %, respectively) and those with and without diabetes (49.3 vs 40.3 %, respectively). This alone shows no clear pattern, which is confirmed by the share of 210+ min walkers in regards to the total number of diseases/chronic conditions. Still, the highest share of 210+ min walkers is reported by respondents with diabetes (49.3 %), kidney problems (47.3 %), and asthma (46.7 %). These data align with recommendations by medical professionals, particularly in terms of improving lung function and blood circulation (9, 39) or blood sugar control (40). In contrast, respondents who had had a heart attack, coronary heart disease, high blood pressure, osteoarthritis, back pain, liver cirrhosis, or depression reported a lower share of 210+ min walkers than those who had not had these diseases/conditions in the last 12 months. The lowest share was reported by respondents with liver cirrhosis (15.6 %), depression (33.9 %), and high blood pressure (39.7 %).

**Table 3:** Share of adults walking ≥210 min a week by chronic diseases/conditions

**Diseases and chronic conditions**	**Self-report of the chronic condition in the past 12 months**	**Share of population walking ≥210 min/week**
Asthma^*^	YES	46.7 %
	NO	40.8 %
Chronic bronchitis, chronic obstructive pulmonary disease or emphysema^*^	YES	41.7 %
	NO	40.9 %
Heart attack or chronic consequences of myocardial infarction^*^	YES	39.1 %
	NO	41.0 %
Coronary heart disease or angina pectoris^*^	YES	40.1 %
	NO	41.0 %
High blood pressure^*^	YES	39.7 %
	NO	41.4 %
Stroke or chronic effects of stroke^*^	YES	43.9 %
	NO	40.9 %
Arthrosis ^*^	YES	38.3 %
	NO	41.1 %
Low back disorder or other chronic back defect^*^	YES	39.6 %
	NO	41.6 %
Neck disorder or other chronic neck defect^*^	YES	39.1 %
	NO	41.5 %
Diabetes^*^	YES	49.3 %
	NO	40.3 %
Allergy, such as rhinitis, eye inflammation, dermatitis, food allergy or other^*^	YES	42.5 %
	NO	40.7 %
Cirrhosis of the liver^*^	YES	15.6 %
	NO	41.1 %
Urinary incontinence or problems in controlling the bladder^*^	YES	41.9 %
	NO	40.9 %
Kidney problems^*^	YES	47.3 %
	NO	40.7 %
Depression^*^	YES	33.9 %
	NO	41.5 %
High blood lipids^*^	YES	41.1 %
	NO	40.9 %
Obesity^*^	YES	43.4 %
	NO	40.8 %
Total sum of diseases/chronic conditions reported in the past 12 months^*^	0	42.4 %
	1 disease/chronic condition	41.3 %
	2 or 3 diseases/chronic conditions	38.4 %
	4 or more diseases/chronic conditions	42.2 %

* p<0.001

Table 4 shows the strength of associations between 210+ min weekly walk and demographic characteristics, health-related behaviours, and chronic diseases/conditions. The binary logistic regression model is statistically significant (χ^2^=142234.142, p<0.001), explains 8.8 % (Nagelkerke R^2^) of the variance, and correctly classifies 62.8 % of cases. Respondents doing mostly heavy or physically demanding work stand out as being 3.04 times more likely to walk 210 min or more a week than those not performing any work-related physical activities. They are followed by respondents spending over 300 min on non-working physical activities a week and those whose work mostly involves walking and moderate activity (2.57 and 2.11 times more likely, respectively). Other characteristics, while being significantly associated with walking 210+ min a week affect this likelihood on a much smaller scale.

**Table 4:** Associations between walking ≥210 min a week and demographic characteristics, health-related behaviour, and chronic diseases/conditions

**Variables**	**Walking ≥210 min a week**	
	**OR (95 % CI)**	**p-value**
**Sex**		
Male	1.17 (1.17–1.18)	p<0.001
Female	Reference	
**Age**		
18–24	Reference	
25–34	1.12 (1.10–1.13)	p<0.001
35–44	1.02 (1.00–1.03)	p=0.011
45–54	0.94 (0.93–0.95)	p<0.001
55–64	1.07 (1.06–1.09)	p<0.001
65–74	1.28 (1.26–1.30)	p<0.001
75+	1.26 (1.23–1.28)	p<0.001
**Degree of urbanisation**		
Cities	Reference	
Towns and suburbs	1.17 (1.16–1.18)	p<0.001
Rural areas	1.18 (1.17–1.19)	p<0.001
**Educational attainment level**		
Lower level of education	Reference	
Medium level of education	1.10 (1.09–1.11)	p<0.001
Higher level of education	1.22 (1.21–1.23)	p<0.001
**NUTS 2 regions (Nomenclature of Territorial Units for Statistics)**		
Pannonian Croatia	1.52 (1.51–1.54)	p<0.001
Adriatic Croatia	1.60 (1.58–1.61)	p<0.001
City of Zagreb	1.56 (1.54–1.58)	p<0.001
Northern Croatia	Reference	
**Smoking**		
Daily smoking	1.08 (1.08–1.09)	p<0.001
Occasional smoking	0.81 (0.80–0.82)	p<0.001
No smoking	Reference	
**Work-related physical activity**		
Mostly sitting or standing	1.03 (0.99–1.07)	p=0.158
Mostly walking or tasks of moderate physical effort	2.11 (2.03–2.19)	p<0.001
Mostly heavy labour or physically demanding work	3.04 (2.92–3.17)	p<0.001
Not performing any working tasks	Reference	
**Time spent on health-enhancing (non-work-related) aerobic physical activity**		
Not performing the activities	Reference	
Less than 60 min	1.22 (1.20–1.24)	p<0.001
60 to less than 150 min	1.26 (1.25–1.27)	p<0.001
150 to less than 300 min	1.79 (1.78–1.81)	p<0.001
300 min and more	2.57 (2.55–2.59)	p<0.001
**Total sum of diseases/chronic conditions reported in the past 12 months**		
0	Reference	
1 disease/chronic condition	0.99 (0.97–1.00)	p=0.145
2 or 3 diseases/chronic conditions	0.86 (0.85–0.86)	p<0.001
4 or more diseases/chronic conditions	1.04 (1.03–1.05)	p<0.001

## CONCLUSION

This study provides valuable insights into the frequency and factors determining healthy walking habits in a representative sample of Croatian adults. It has some limitations, though. Firstly, being a cross-sectional study, it cannot establish the cause-and-effect relationships between specific variables and regular walking. Given the intriguing differences between certain diseases, this limitation could be overcome by investigating the origins of these differences in a longitudinal study. For instance, it would be beneficial to explore whether not walking partly contributes to the development of liver cirrhosis, depression, or hypertension, or it is the consequence of these diseases. Secondly, while this study does establish that some demographic, behavioural, and health factors are independently associated with regular walking, our model explains only a small share of the total variance. This implies that there are other factors associated with regular walking that should be more closely investigated, such as environmental and psychological factors. Given the well-established health benefits of walking and the relatively low prevalence of regular walking among adults in Croatia, it is imperative to plan further studies built upon our findings. These studies could inform public health policies to encourage walking and, more to the point, promote healthy lifestyles.

Our findings also highlight the need to raise awareness among specific population groups and change their habits, which can be achieved by concerted cooperation between stakeholders in politics, healthcare, and sports. One way is to strengthen local communities and encourage them to set up accessible infrastructure for all age groups (41,42,43) and launch initiatives aimed at popularising walking, such as the Walking Towards Health initiative (8), which has been implemented in local communities across the country for several years.
